# Synthesis and characterization of a new photoinduced switchable β-cyclodextrin dimer

**DOI:** 10.3762/bjoc.10.304

**Published:** 2014-12-04

**Authors:** Florian Hamon, Claire Blaszkiewicz, Marie Buchotte, Estelle Banaszak-Léonard, Hervé Bricout, Sébastien Tilloy, Eric Monflier, Christine Cézard, Laurent Bouteiller, Christophe Len, Florence Djedaini-Pilard

**Affiliations:** 1 Université de Picardie Jules Verne, Laboratoire de Glycochimie – Antimicrobiens et Agroressources, LG2A FRE CNRS 3517, F-80039 Amiens, France; 2 Université d’Artois, Unité de Catalyse et de Chimie du Solide (UCCS), CNRS, UMR 8181, Rue Jean Souvraz, SP 18, F-62307 Lens, France; 3 Transformations Intégrées de la Matière Renouvelable, TIMR EA4295 UTC/ESCOM, F-60200 Compiègne, France; 4 Sorbonne Universités, UPMC Université Paris 06, CNRS, UMR 8232, IPCM, Chimie des Polymères, F-75005, Paris, France; 5 CNRS, UMR 8232, IPCM, Chimie des Polymères, F-75005, Paris, France

**Keywords:** azobenzene, cyclodextrins, inclusion complex, photoisomerization, switchable binding behavior

## Abstract

This paper reports an efficient preparation of bridged bis-β-CD AZO-CDim **1** bearing azobenzene as a linker and exhibiting high solubility in water. The photoisomerization properties were studied by UV–vis and HPLC and supported by ab initio calculations. The *cis/trans* ratio of AZO-CDim **1** is 7:93 without irradiation and 37:63 after 120 min of irradiation at 365 nm; the reaction is reversible after irradiation at 254 nm. The photoinduced, switchable binding behavior of AZO-CDim **1** was evaluated by ITC, NMR and molecular modeling in the presence of a ditopic adamantyl guest. The results indicate that AZO-CDim **1** can form two different inclusion complexes with an adamantyl dimer depending on its photoinduced isomers. Both cavities of *cis*-AZO-CDim **1** are complexed simultaneously by two adamantyl units of the guest forming a 1:1 complex while *trans*-AZO-CDim **1** seems to lead to the formation of supramolecular polymers with an *n*:*n* stoichiometry.

## Introduction

α-, β- or γ-Cyclodextrins (CDs) are cyclic oligosaccharides composed of 6, 7 or 8 α-D-1,4 glucopyranose moieties, respectively. They are natural compounds produced from starch by the reaction of 4-α-glucanotransferases [1]. Their toroidal shape, with C6-primary hydroxy groups on the narrow rim and C2 and C3 secondary groups on the wider rim, enables encapsulation of hydrophobic molecules inside their cavity. Since the 1950s, it has been demonstrated that CDs can form non-covalent-force complexes in water due to their unique spatial arrangement. In particular, β-cyclodextrin (β-CD) is known to form supramolecular inclusion complexes with molecules, and such inclusion usually enhances the solubility of water-insoluble substances [1–4]. Pharmaceutical companies already use these cyclodextrins or their derivatives in their formulations [5–6]. In fact, they have a well-defined structure, low toxicological or pharmacological activity, and good solubility in water. For example, the inclusion of active substances in CDs can reduce their undesirable storage or metabolism degradation, which has led research on CDs to focus on controlled drug delivery [4]. In the food industry, CDs enable the fixation or retention of volatile flavors, as well as the removal of undesirable flavors from food [7–8].

In comparison with CD monomers, bridged bis-cyclodextrins can improve the original binding ability of native CDs through the cooperative binding of both cavities located close to the guest molecules [9–10]. These cyclodextrins linked by ester [11], thioether [12–16], urea [17–19], or triazole [20] moieties have been previously described. In addition, aromatic azobenzenes are excellent candidates as molecular switch linkers as they have two forms, namely *cis* (*Z*) and *trans* (*E*) isomers, which can be interconverted by both photochemical and thermal means [21]. This transformation by external stimuli induces a molecular movement and a significant geometric change [22–23]. CDs and azobenzene derivatives can form inclusion complexes controlled by photoisomerization of the guests and this property has been widely applied to molecular shuttles, motors, information storage [24–25] and catalysis [26].

Some examples of azobenzene-linked CD dimers can be found in the literature but they generally suffer from arduous purification steps and very low yields [27–28]. As an exception, Vargas et al. [29] described the synthesis of 1,2,3-triazole-linked azobenzene-cyclodextrin derivatives producing rather good yields but the photoisomerization and inclusion complex properties were not investigated. Here, we report an efficient preparation of a new bis-β-CD with azobenzene dicarboxylate and the influence of photoisomerization of the linker on the conformation and binding behavior of the CD dimer.

## Results and Discussion

**Scheme 1:**
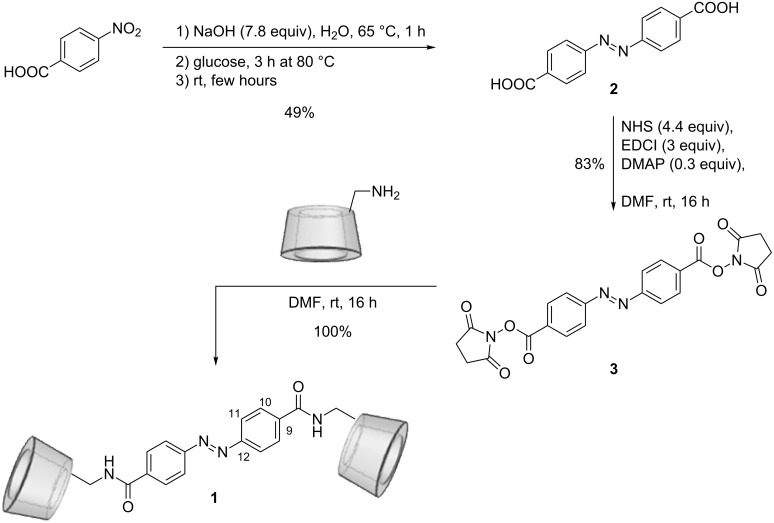
Synthesis pathway of the dimer AZO-CDim **1**.

The AZO-CDim **1** synthesis was performed as follows: 4,4’-azobenzenedicarboxylic acid was first obtained by reductive coupling of 4-nitrobenzoic acid with a yield of 49% (Scheme 1) [30]. Then, the carboxylic groups were activated by *N*-hydroxysuccinimide (NHS) and condensed with mono-6-amino-6-deoxy-β-cyclodextrin (β-CD-NH_2_) [31] in anhydrous DMF at room temperature. Flash chromatography (C_18_ column, H_2_O/MeOH 90:10 to 10:90 v/v in 20 min) afforded pure AZO-CDim **1** with 62% yield.

It should be noted that AZO-CDim **1** exhibits surprisingly high solubility in water, reaching 220 mM at 293 K, even though the linker is hydrophobic and the solubility of β-CD is only 16 mM under the same conditions. Concentration-variable NMR analysis revealed a strong upfield shift and a broadening of the proton signals. No significant chemical shift variations were observed at concentrations below 1 mM, which is consistent with a critical aggregation concentration of around 1·10^−3^ M.

**Figure 1:**
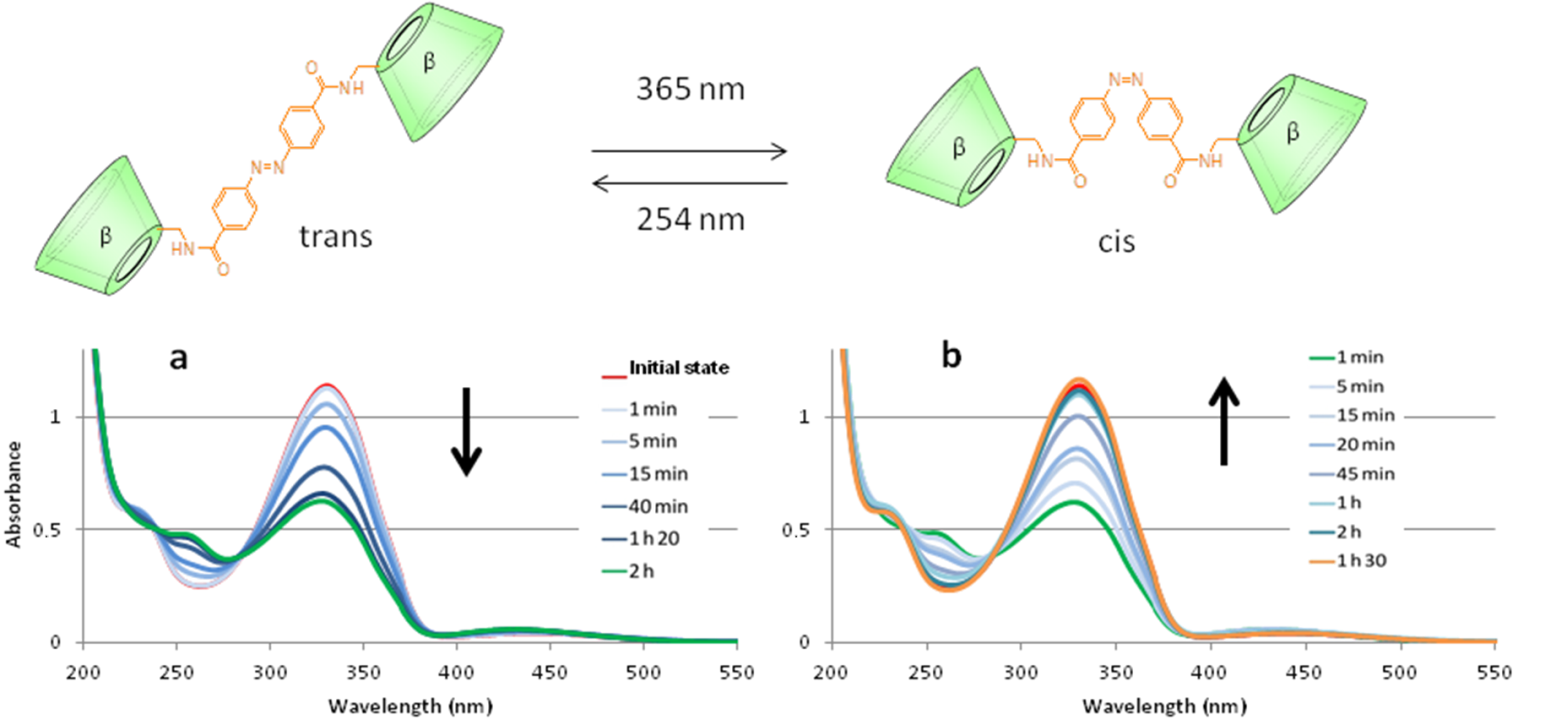
Overlaid UV spectra of the irradiation of AZO-CDim **1** (a) from 0 to 120 min at 365 nm and then (b) from 0 to 90 min at 254 nm; (*c* = 10^−4^ M, water, 6 W lamp).

The UV–vis absorption spectrum of azobenzene presents three characteristic absorption bands (250, 320 and 450 nm) corresponding to π–π* and n–π* electronic transitions, respectively. For the *trans* isomer, the absorption band π–π* at 320 nm is very intense while the other two bands (π–π*) at 250 nm and (n–π*) at 420 nm are much weaker. For the *cis* isomer, the absorption band π–π* is shifted slightly to a shorter wavelength and is significantly less intense at 320 nm. Because the n–π* transition is possible in the *cis* isomer, this band increases in intensity [22–23]. A molecular switch is based on the light-induced, reversible transformation of chemical species between two molecular states with different absorption spectra. Thus, the *trans*/*cis* isomerization can be reversibly controlled through UV light irradiation as depicted in Figure 1. As shown in Figure 1a, when a sample containing AZO-CDim **1** in pure water at room temperature was UV irradiated at 365 nm, it switched from its *trans* to its *cis* form resulting in a marked change in the UV–vis spectra. As the irradiation continued, the absorption band at around 320 nm gradually decreased while the bands at 420 nm and 250 nm slightly increased. This change is clearly due to the simple, but partial, isomerization of the azo groups from the *trans* photoisomer to the *cis* photoisomer [21]. The maximum isomerization yield was obtained after 120 min of irradiation at 365 nm.

**Figure 2:**
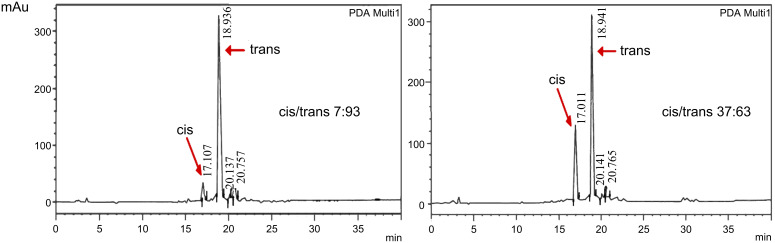
HPLC quantification of the *cis*/*trans* ratio of AZO-CDim **1** before irradiation (left) and after irradiation at 365 nm (2 h, 6 W lamp) (right).

The reaction is reversible and when irradiated at 254 nm (Figure 1b), the *cis* isomer of AZO-CDim **1** gradually returned to its *trans* form, and the maximum isomerization yield was obtained after 90 min of irradiation. Both isomers could be separated by HPLC (Dionex, H_2_O/MeCN 90:10) and the *cis*/*trans* ratio of AZO-CDim **1** before irradiation (7:93) and after 2 h of irradiation (37:63) at 365 nm was determined (Figure 2). Although each isomer could not be obtained in pure form, as is often the case for many azoderivatives [32], the isomerization efficiency is better than the *cis*/*trans* ratio of 14:86 after irradiation described by Liu [27].

**Figure 3:**
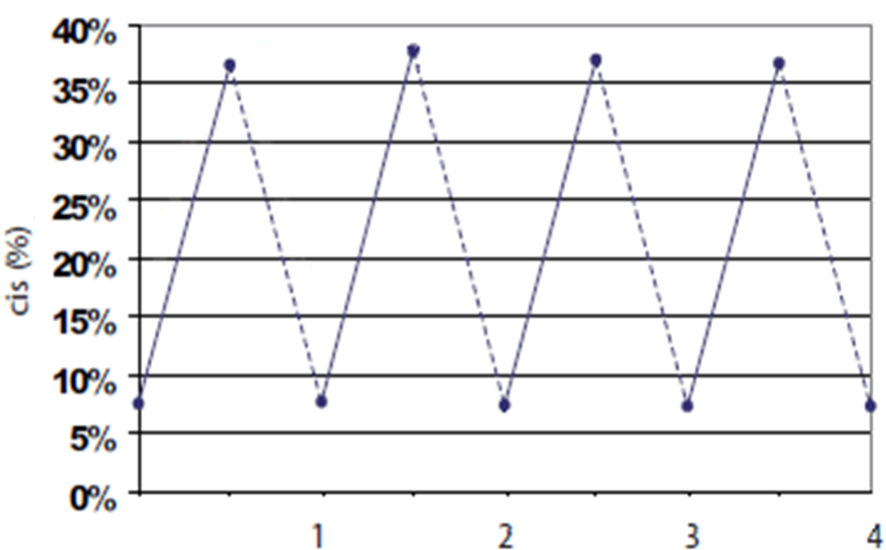
Percentage of *cis* isomer of AZO-CDim **1** produced during photoisomerization cycles (*c* = 10^−4^ M, water). A cycle consists of irradiation at 365 nm for 2 h followed by irradiation at 254 nm for 1 h.

Both isomers of AZO-CDim **1** have an appreciable resistance to fatigue thus the irradiation cycle could be carried out several times without causing side effects, as shown in Figure 3.

**Figure 4:**
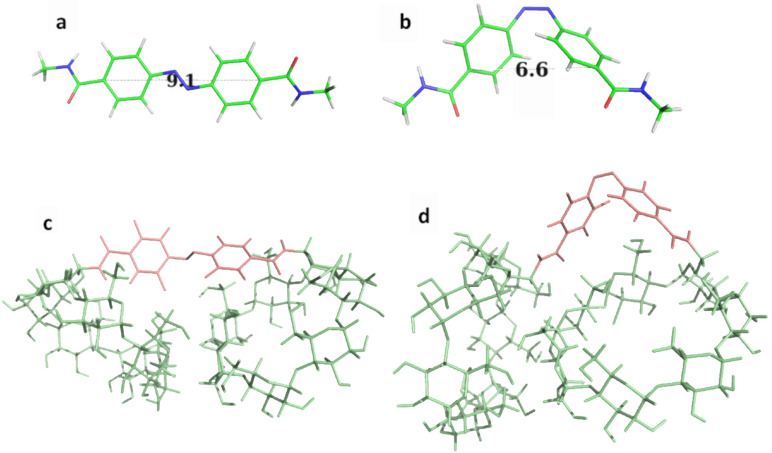
Representation of the most stable structures obtained for the azobenzene linker (a) for the *trans* configuration and (b) for the *cis* one and for the AZO-CDim **1** systems (c) for the *trans* configuration and (d) for the *cis* one.

Ab initio calculations were also performed but the *cis*/*trans* transition was not observed since molecular modeling methods are unable to break bonds. In order to collect data on this phenomenon, the two configurations of the system had to be taken into account separately. Thus, ab initio calculations were performed on the two configurations of the azobenzene linker, the so-called 4,4’-bis(*N*-methylcarboxamide)azobenzene linker, to determine the structures of minimal energy (Figure 4a and Figure 4b). Once optimized, the measured C4–C4’ distances were 9.1 and 6.6 Å for the *trans* and *cis* configurations, respectively, and the C–N=N–C dihedral angles were 180° and −10°, respectively. These results are comparable to those obtained by Koshima et al. [33–34] on crystal structures where intermolecular packing effects might be important. From these calculations, the geometrical force field parameters needed for molecular dynamics simulations were derived.

Molecular dynamics simulations performed on the two configurations of AZO-CDim **1** highlighted the rigidity of the linker, which governs the relative position of the two CD cavities. The trajectories, corresponding to 50,000 snapshots, were clustered into thirty representative conformations. The most stable structure of these thirty representative conformations for each configuration is shown in Figure 4c and Figure 4d. It should be kept in mind that although the linker is quite rigid, the two CD cavities can rotate and move around the azobenzene axis. Throughout the simulations, the C4–C4’ distances and the C–N=N–C dihedral angles did not fluctuate much. The C–C average value was 8.9 ± 0.1 and 5.8 ± 0.3 Å and the C–N=N–C dihedral angle was 175.1 ± 4.8 and −6.3 ± 5.4° for the *trans* and *cis* configurations, respectively.

**Figure 5:**
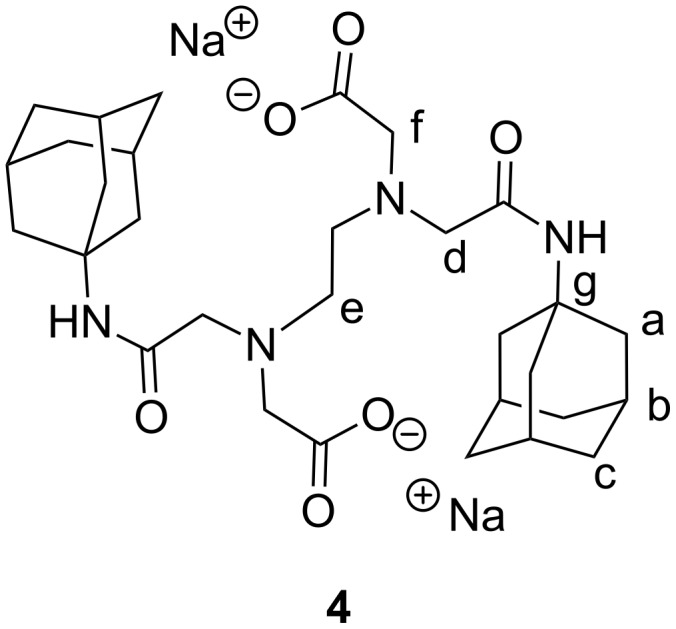
Structure of the ditopic guest ADAdim **4**.

In these conditions, using azobenzene as a linker between two β-CD can lead to a modulation of the inclusion properties, such as a cooperative effect. The cavity of each CD is available to form an inclusion complex with a hydrophobic guest molecule. Among these, adamantane is known to be an excellent guest for the β-CD cavity, with an association constant *K*_a_ ranging from 2·10^4^ M^−1^ to 4·10^4^ M^−1^ [35]. This is because the adamantyl residue fits perfectly inside the β-CD cavity. In this present work, we investigated how the affinity between the dimer of adamantane and switchable AZO-CDim **1** may be influenced by the *cis*/*trans* ratio of the host molecule. For this purpose, we synthesized the adamantyl dimer EDTA bis-1-aminoadamantyldiamide disodium salt, ADAdim **4**, as described by Vasquez Tato et al. [36] (Figure 5)*.* These authors showed that the single interaction between one binding site of the ditopic guest ADAdim **4** and one binding site of a particular β-CD dimer, bearing a terephthalic acid linker, was independent of the number of binding sites, that is, no cooperative effect was observed and a supramolecular polymer was formed.

**Figure 6:**
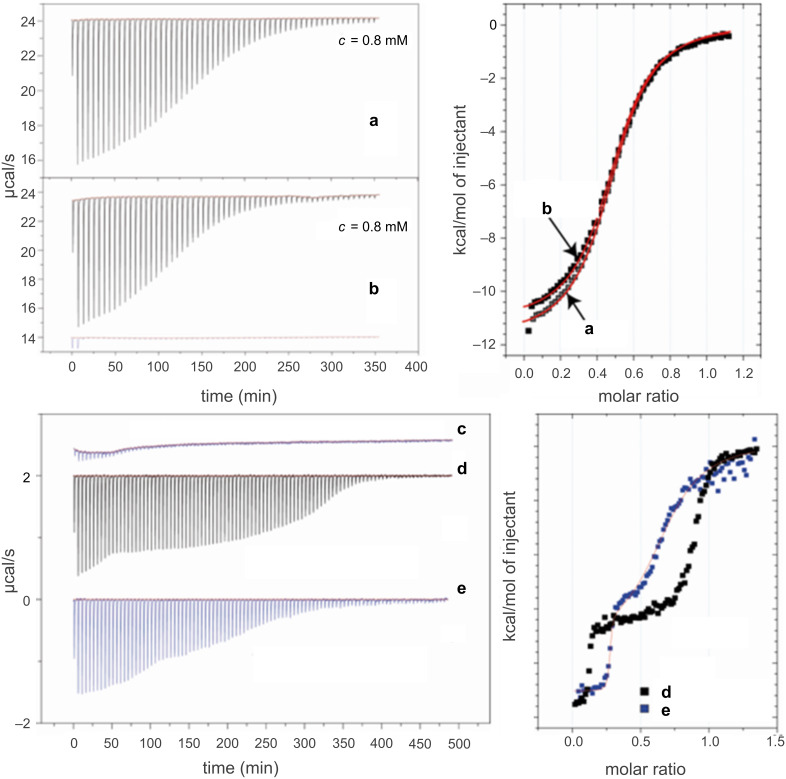
Titration of (a) β-CD (*c* = 0.8 mM) and (b) β-CD-NH_2_ (*c* = 0.8 mM) by ADAdim **4** (*c =* 4 mM). (c) Dilution of ADAdim **4** (*c* = 0.6 mM) in water at 298 K. Titration of AZO-CDim **1** (*c* = 0.1 mM) by ADAdim **4** (*c* = 0.6 mM) (d) before irradiation at 298 K and (e) after irradiation in water at 365 nm for 120 min at 278 K.

ITC is one of the most interesting methods to characterize the interaction of CDs with guests in solution [37–38]. It enables the enthalpy, entropy and equilibrium constants involved in complexation processes to be determined in a single experiment. Moreover, the guest:host molar ratio (i.e., the stoichiometry of the complex) can be measured. First, enthalpies of dilution of the monotopic hosts β-CD and β-CD-NH_2_, the ditopic host AZO-CDim **1** and the guest ADAdim **4** were measured in separate experiments to determine the maximum concentration to use for the ITC experiments. Enthalpies of dilution of β-CD and β-CD-NH_2_ were negligible over a broad concentration range, whereas enthalpies of dilution of AZO-CDim **1** were negligible only for concentrations lower than 1 mM, which is in agreement with NMR data. As observed by Vásquez Tato and coworkers [39], ADAdim **4** can be considered as a surfactant. However, by using a maximum concentration of 4 mM in the ITC experiment, the effect of any heat resulting from a deaggregation process can be avoided. First, the interactions between β-CD or β-CD-NH_2_ and ADAdim **4** (added to the CD solution) were studied by ITC. After integrating the heat signal as a function of the molar ratio between the guest and the host, the isotherm was fitted to the one-site binding model as shown in Figure 6. The average values for the thermodynamic parameters are given in Table 1.

**Table 1:** Thermodynamic parameters deduced from ITC experiments for the different host–guest systems studied (temperature 298 K in water).

Host	*n*^a^	*K*_a_ (M^−1^)	Δ*H*^0^ (kJ·mol^−1^)	Δ*H*^0^ guest site (kJ·mol^−1^)	*T*Δ*S*^0^ (kJ·mol^−1^)	Δ*G*^0^ (kJ·mol^−1^)

β-CD^b^	0.50 (1:2)	(4.6 ± 0.2)·10^4^	−45.3 ± 0.3	−22.7 ± 0.2	−9.3 ± 0.2	−13.4 ± 0.1
β-CD-NH_2_^b^	0.43 (1:2)	(5.0 ± 0.2)·10^4^	−49.0 ± 0.2	−24.5 ± 0.1	−11.1 ± 0.2	−13.4 ± 0.1
AZO-CDim **1**^c^ without irradiation	0.13 (0.07:1)	(8 ± 0.2)·10^8^	−95 ± 0.7	−47.5 ± 0.4	−17.1 ± 0.8	−27.7 ± 0.4
	0.77 (0.93:1)	(8 ± 0.2)·10^5^	−67 ± 0.3	−33.5 ± 0.2	−13.4 ± 0.4	−18.4 ± 0.2
AZO-CDim **1**^d^ after irradiation	0.27 (0.37:1)	(8 ± 0.2)·10^8^	−95 ± 0.8	−47.5 ± 0.4	−25.2 ± 0.8	−21.9 ± 0.4
	0.44 (0.63:1)	(8 ± 0.2)·10^5^	−67 ± 0.4	−33.5 ± 0.2	−23.8 ± 2.4	−13.7 ± 1.2

*n*^a^: guest:host molar ratio in the complex. The values in parentheses are theoretical values for the formation of complexes with all the binding sites of both guest and host occupied. ^b^Values corresponding to the model of one set of sites. ^c^Values corresponding to the model of two sets of sites. ^d^Values corresponding to the two sets of sites model after irradiation at 365 nm, for 120 min at 278 K. The units of the experimental enthalpy value are kJ·mol^−1^ of the titrating species (i.e. the guest species).

For β-CD and β-CD-NH_2_, the experimental *n* values (0.50 and 0.43, respectively) for the complexes correspond to one ADAdim **4** for two cyclodextrins (stoichiometry 1:2). Considering the slightly different experimental conditions, all the data obtained are in very good agreement with the literature [36,39]. The same study was performed with AZO-CDim **1** exhibiting a *cis*/*trans* ratio of 7:93 and the experimental values are presented in Table 1. As depicted in Figure 6d, two jumps can be observed in the calorimetric titration curves revealing two independent interactions. The experimental curve was well-fit to the two-sites binding model and the following results were achieved: the first jump corresponds to a very strong interaction between a small fraction of AZO-CDim **1** and ADAdim **4** with *n* = 0.13 and *K*_a_ = 8·10^8^ M^−1^. The second jump corresponds to a weaker interaction between a major fraction of AZO-CDim **1** and ADAdim **4** with *n* = 0.77 and *K*_a_ = 8·10^5^ M^−1^. Assuming a 1:1 stoichiometry in both cases, the first jump involves 14% of the mixture and the second 86% in relatively good agreement with the *cis*/*trans* ratio measured by HPLC (7:93). It is therefore tempting to assign the first jump to the complexation between ADAdim **4** and the *cis* isomer of AZO-CDim **1** and the second jump to the complexation between ADAdim **4** and the *trans* isomer of AZO-CDim **1**.

To confirm this hypothesis, the same ITC study was performed after UV irradiation at 365 nm for 120 min (Figure 6e). To improve the stability of *cis*-AZO-CDim **1**, the titration was carried out at 278 K under the same experimental conditions as previously performed. The average values for the thermodynamic parameters are summarized in Table 1. Again, two jumps can be observed in the enthalpogram (Figure 6d), the stoichiometry of which has been shifted due to the UV irradiation. Neither the 20 °C difference nor the changing stoichiometry is thought to greatly affect the formation constants of the complexes. We therefore attempted a fit of both experimental curves with the same values for association constants and enthalpies. The following parameter values afforded a reasonable fit: *K*_1_ = 8·10^8^ L·mol^−1^, Δ*H*_1_ = −95 kJ·mol^−1^, *K*_2_ = 8·10^5^ L·mol^−1^, Δ*H*_2_ = −67 kJ·mol^−1^, with stoichiometries *n*_1_ = 0.26 and *n*_2_ = 0.42 (vs experimental stoichiometries *n*_1_ = 0.27 and *n*_2_ = 0.44) after irradiation. Assuming a 1:1 stoichiometry in both cases, the first jump involves 38% of the mixture and the second 62%, again matching the *cis*/*trans* ratio measured by HPLC (37:63). This unambiguously proves that the two jumps detected by ITC correspond to the complexation of both isomers of AZO-CDim **1**.

Interestingly, the association constants measured for the ditopic host (about 10^9^ M^−1^ for the *cis* isomer and 10^6^ M^−1^ for the *trans* isomer) are orders of magnitude larger than the association constant for the monotopic β-CD (*K*_a_ = 5·10^4^ M^−1^). This means that the complexation is highly cooperative, particularly in the case of the *cis* isomer. Although it was not demonstrated, such an additional interaction could explain why the association constant between the *trans* isomer of the ditopic host AZO-CDim **1** and the ditopic guest ADAdim **4** is significantly larger than that between β-CD and ADAdim **4**. This, in turn, hints at particularly well matched conformations, as shown by the molecular simulation (see below).

**Figure 7:**
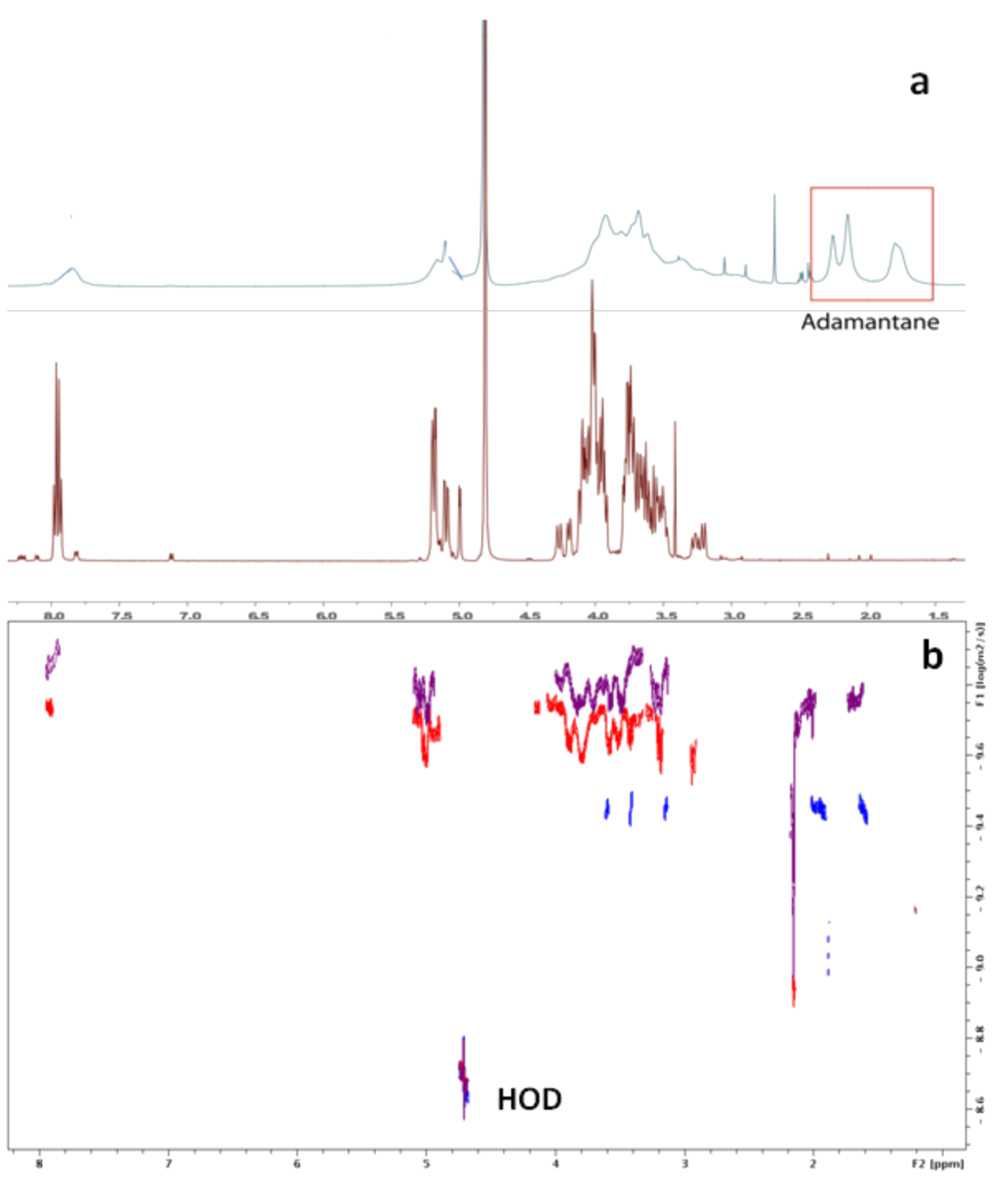
(a) ^1^H NMR spectra of AZO-CDim **1** (500 MHz, D_2_O, 2.5 mM) in the absence (bottom) and presence of ADAdim **4** (2.5 mM, top); (b) DOSY spectra of AZO-CDim **1** (red), ADAdim **4** (blue) and an equimolecular mixture of both (purple), (1 mM, D_2_O).

The ^1^H NMR spectra of AZO-CDim **1** with a *cis*/*trans* ratio of 7:93 were obtained in the absence or presence of an equimolar concentration of ADAdim **4** (Figure 7a). As previously stated, each *cis* and *trans* isomer could not be isolated in pure form, complicating the NMR study. In the presence of the ditopic guest ADAdim **4**, a strong broadening of all signals was observed, indicating the presence of large objects in solution. A ROESY experiment was also carried out and although the presence of cross-correlation peaks between protons of AZO-CDim **1** and ADAdim **4** supports an inclusion complex, the strong signal broadening impeded any assignment (data not shown).

Diffusion-ordered spectroscopy (DOSY) is a solution-based NMR method used to discriminate signals arising from different species by their diffusion rates. This method is very helpful and convenient for characterizing molecular aggregates or inclusion complexes [40]. The diffusion coefficient (*D*) is directly related to the molecular mass of the observed species in solution. In other words, when the molecular mass increases, the diffusion rate decreases. The DOSY spectra for ADAdim **4,** AZO-CDim **1,** and an equimolecular mixture of both were recorded in D_2_O (Figure 7b) and the measured *D* values were 3.540·10^−10^ m^2^·s^−1^, 2.293·10^−10^ m^2^·s^−1^ and 1.33·10^−10^ m^2^·s^−1^, respectively. The *D* value of ADAdim **4** is smaller than that of AZO-CDim **1**, in accordance with their molecular masses. It is usually assumed that the *D* value of an inclusion complex is the same as that of the host molecule alone [40], however, this was not observed in our case, which is in agreement with the formation of larger objects in solution.

**Figure 8:**
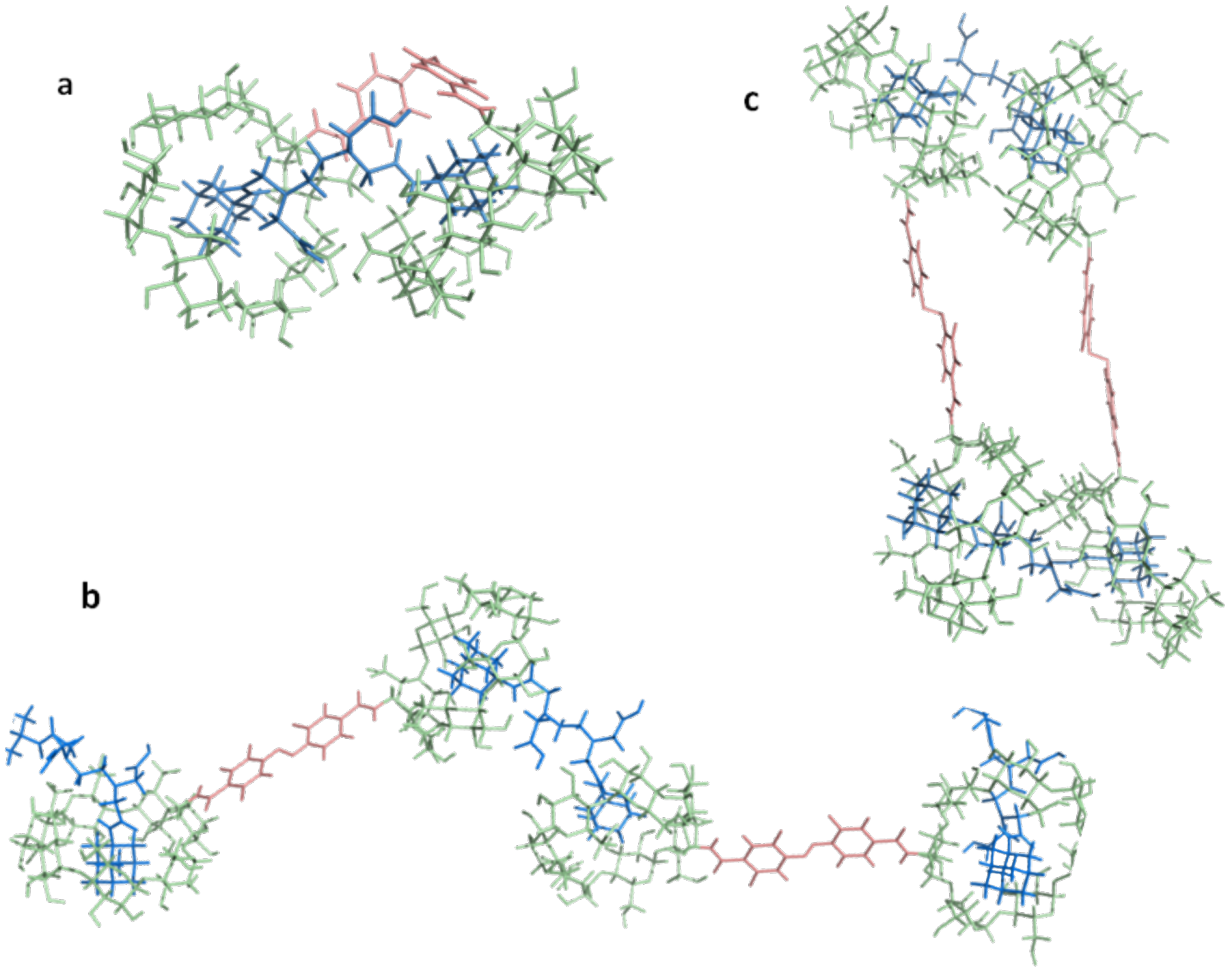
Proposed structures of inclusion complexes with the ditopic host AZO-CDim **1** and the ditopic guest ADAdim **4** after minimization by molecular modeling methods: (a) 1:1 chelate-type complex with AZO-CDim **1** in its *cis* configuration; linear (b) and cyclic (c) supramolecular polymers with AZO-CDim **1** in its *trans* configuration.

The experimental data strongly suggests that AZO-CDim **1** is a switchable host which forms two different inclusion complexes with this ditopic guest. The structural analyses of the molecular dynamics trajectories of the two configurations of the AZO-CDim **1** systems enable us to draw some conclusions as to how the adamantyl units could be contained in one or both cavities. To further support our assumptions, the corresponding systems were built and minimized. The following main conclusions can be drawn. For the first, in the *cis* configuration, both cavities of the ditopic host AZO-CDim **1** are available for complexation and their orientation favors the simultaneous inclusion of both adamantyl units of ADAdim **4**, forming a 1:1 chelate-type complex depicted in Figure 8a. The chelate effect has been extensively studied by Breslow et al. [41–42] among others and a higher stability constant is expected due to the strong cooperative effect. Regarding the second main conclusion, the size and rigidity of the linker in AZO-CDim **1** prevent the *tran*s configuration from forming ditopic 1:1 complexes upon complexation with only one molecule of ADAdim **4**. Nevertheless, the two cavities remain available for complex formation through their wider rim with two adamantyl units belonging to two different ADAdim **4** molecules, leading to the formation of supramolecular polymers with an *n*:*n* stoichiometry. This situation has already been encountered in the complex of ADAdim **4** and a β-CD dimer bearing a terephthalic moiety as the linker [36]. At this stage, based on the molecular dynamics study, at least two supramolecular polymers can be considered: the first is linear, as often described in the literature [43–44] (Figure 8b), and the second is cyclic (Figure 8c). Furthermore, it is possible that such linear or cyclic polymers are aggregated into larger objects stabilized by hydrogen bonds between cyclodextrin moieties and by π-stacking interactions between azobenzene linkers.

Finally, according to the computational data, the complexes of AZO-CDim **1** with ADAdim **4** are further stabilized by intramolecular interactions between CD subunits that are more favorable in the *cis* than in the *trans* configurational arrangement, which is in agreement with the ITC data.

## Experimental

### Materials and methods

All solvents were used as purchased, unless otherwise noted. All starting materials were used without purification. β-CD was purchased from Roquette Frères (Lestrem, France) and β-CD-NH_2_ was synthesized as previously described [31] or purchased from Biocydex (Poitiers, France). Analytical TLC was performed using Silica Gel 60 F_254_ plates (Merck, Germany). Eluents were mixtures of dichloromethane/methanol or cyclohexane/ethyl acetate. Ratios are specified in each case in the experimental section. Products were illuminated under UV light (λ = 254 nm) followed by charring with vanillin/H_2_SO_4._

UV analyses were performed on a UV–vis Cary VARIAN spectrophotometer coupled with an optic fiber. A 6 Watt mercury lamp was used (λ = 365 nm) for the irradiation of aqueous AZO-CDim **1** solutions (*c* = 10^−4^ M).

Stepwise control of the reactions was readily achieved by ESIMS in the positive ion mode using a ZQ 4000 quadrupole mass spectrometer (Waters-Micromass, Manchester, UK). High resolution electrospray mass spectra (HRMS–ESI) operated in the positive ion mode were obtained on a Q-TOF Ultima Global instrument (Waters-Micromass, Manchester, UK). Data acquisition and processing were performed with MassLynx 4.0 software. High resolution mass spectra were recorded in the positive mode on a ZabSpec TOF (Micromass, UK) tandem hybrid mass spectrometer with EBETOF geometry. The compounds were individually dissolved in a 1:1 water/CH_3_CN mixture at a concentration of 10 μg·cm^−3^ and then infused into the electrospray ion source at a flow rate of 10 mm^3^·min^−1^ at 333 K. The mass spectrometer was operated at 4 kV while scanning the magnet over a typical range of 4000–100 Da. The mass spectra were collected as a continuum profile data. Accurate mass measurement was achieved using polyethylene glycol as the internal reference mass with a resolving power set to a minimum of 10,000 (10% valley).

NMR experiments were performed at 300.13 and 600.13 MHz using Bruker AVANCE DPX300 and AVANCE 600 spectrometers equipped with a Z-gradient unit for pulsed-field gradient spectroscopy. Me_4_Si was used as an external standard and calibration was performed using the signal of the residual protons or of the carbon of the solvents as a secondary reference. Measurements were performed at 300 K with careful temperature regulation. The length of the 90° pulse was approximately 7 μs. 1D NMR data spectra were collected using 16K data points. 2D experiments were run using 1K data points and 512 time increments. The phase-sensitive (TTPI) sequence was used and processing resulted in a 1K·1K (real-real) matrix. The DOSY experiments were performed using the ledbpgp2s sequence from the Bruker library, with stimulated echo, longitudinal eddy current compensation, bipolar gradient pulses and two spoil gradients using 16 different gradient values varying from 2 to 95% of the maximum gradient strength. A 100 ms diffusion time and a 2.2 ms gradient length were used.

Isothermal Titration Calorimetry (ITC) was performed using a VP-ITC microcalorimeter at 298 K or 278 K in pure water. Briefly, titration was carried out with 60 injections of 5 µL every 6 min. Control experiments were performed by the dilution of the guest solution in water and showed small heats of dilution. Thus, these results were subtracted from each titration to remove guest heats of dilution. The experimental data were fitted to a theoretical titration curve using Origin 7.0, Microcal software with the one set or two sets of sites models. During this fitting, enthalpy (Δ*H*), stoichiometry (*n*) and association constants (*K*_a_) were adjustable parameters.

### Molecular modeling

Initial geometries of the β-cyclodextrin dimers studied in this work were built using the LEaP program from the AmberTools 1.4 distribution, following the strategy and methodology previously established [45]. Except for the linker, the CD fragments were taken from the R.E.DD.B. database [46] under project F-85 (http://q4md-forcefieldtools.org/REDDB/). Both the linker fragment and the cation were defined and parameterized according to the strategy previously developed using the RED program [47] along with the RED server [48].

Molecular dynamics (MD) simulations were performed using the SANDER module of the AMBER10 program suite to perform MD simulations on the aforementioned complexes [49]. The systems were solvated in a truncated octahedral box with a buffer distance of 10.0 Å. The q4md-CD force field parameters were used to model the β-CD systems [45]. The parameters used for water were taken from the TIP3P model [50]. Classic MD simulations of 50 ns were then performed using the NPT ensemble at a pressure of 1 atm and a temperature of 300 K. In order to obtain representative ensembles of conformations for the two bis-CD systems, molecular configurations from MD trajectories were clustered.

Ab initio calculations were performed with the Gaussian 09 program [50] to perform quantum chemical calculations. The structures corresponding to the two configurations of the linker were optimized at the B3LYP level of theory using the 6-31+G* basis set.

### Synthesis

4,4’-Dicarboxyazobenzene (**2**): *p*-nitrobenzoic acid (7.72 g, 46.2 mmol, 1 equiv) and sodium hydroxide (14.24 g, 356 mmol, 7.8 equiv) in 100 mL of water were heated at 338 K for 1 h. Then, 120 mL of 60 wt % glucose solution in water was added dropwise in two portions separated by 1 h of stirring. The mixture was heated at 353 K for 3 h and dropped into a large crystallizer where a precipitate appeared on the surface after several hours. The solid was filtered, dissolved in hot water, acidified with 100 mL of acetic acid, the precipitate filtered again and dried under reduced pressure. 4,4’-Dicarboxyazobenzene (**2**) was obtained as a pink solid (*m* = 6.11 g) with a yield of 49%. The analyses are in full agreement with the literature [30]. Mp > 523 K (dec); ^1^H NMR (DMSO-*d*_6_, 300.13 MHz) δ 8.16 (d, *J* = 8.4 Hz, 4H), 8.01 (d, *J* = 8.4 Hz, 4H) ppm; ^13^C NMR (DMSO-*d*_6_, 75.77 MHz) δ 166.9, 154.3, 133.7, 130.9, 123.0 ppm; ESIMS (*m*/*z*): [M − H]^−^ calcd for C_14_H_9_N_2_O_4_, 269.1; found, 268.9.

4,4’-Dicarboxyazobenzene bis(*N*-hydroxysuccinimide ester) (**3**): Compound **2** (1.0 g, 3.70 mmol, 1 equiv), *N*-hydroxysuccinimide (1.87 g, 16.28 mmol, 4.4 equiv) and DMAP (90 mg, 0.74 mmol, 0.2 equiv) were dissolved in 10 mL of DMF at room temperature. After 10 min of stirring, EDCI (2.13 g, 11.1 mmol, 3 equiv) was added, then the solution was stirred at room temperature for 16 h under an inert atmosphere. The mixture was extracted by 100 mL of DCM and 100 mL of HCl (0.1 M). The aqueous phase was extracted twice with 50 mL of DCM. Organic phases were combined, dried over Na_2_SO_4_ and purified over a plug-in of silica with DCM/MeOH (99:1 v/v) as eluent to obtain **3** as a red solid (*m* = 1.41 g) with a yield of 83%. Mp > 523 K (dec); ^1^H NMR (CDCl_3_, 300.13 MHz) δ 8.33 (d, *J* = 8.4 Hz, 4H), 8.07 (d, *J* = 8.4 Hz, 4H), 2.95 (s, 8H) ppm; ^13^C NMR (CDCl_3_, 75.77 MHz) δ 169.3, 161.4, 155.9, 132.0, 127.7, 123.6, 25.9 ppm; HRMS–ESI (*m*/*z*): [M + Na]^+^ calcd for C_22_H_16_N_4_O_8_Na, 487.0866; found, 487.0876.

*N*,*N*’-Bis[6^I^-deoxy-β-cyclodextrin-6^I^-yl]carboxamide-4,4’-azobenzene, AZO-CDim (**1**): β-CD-NH_2_ (2.02 g, 1.78 mmol, 2 equiv) and **3** (404 mg, 0.87 mmol, 1 equiv) were dissolved in 5 mL of dried distilled DMF. After 16 h of stirring at room temperature, the mixture was concentrated and the product precipitated by addition of acetone, then dried under reduced pressure. The product **1** was obtained as an orange powder (*m* = 4.44 g) with a quantitative yield and an HPLC purity over 98%. Then, the product was purified by flash chromatography (20 min, H_2_O/MeOH from 90:10 to 10:90 (v/v), 40 mL·min^−1^) to afford compound **1** (2.75 g, 62%). Mp 423 K (dec); ^1^H NMR (D_2_O, 600.13 MHz) δ 7.94 (d, ^3^*J*_H10–H11_ = 8.1 Hz, H_10,_ 4H), 7.90 (d, ^3^*J*_H11–H10_ = 8.1 Hz, H_11_, 4H), 4.97–5.17 (m, H_1_^I–VII^, 14H), 3.14–4.25 (m, H_2_^I–VII–^H_3_^I–VII–^H_4_^I–VII–^H_5_^I–VII^-H_6_^I–VII^–H_6_^I–VII^, 84H) ppm; ^13^C NMR (D_2_O, 150.76 MHz) δ 168.19 (C=O), 153.56 (C_12_), 135.35 (C_9_), 128.21 (C_10_), 122.94 (C_11_), 101.28–101.93 (C_1_^I–VII^), 83.69 (C_4_^I^), 80.26–81.05 (C_4_^II–VII^), 71.63–73.54 (C_2_^I–VII^–C_3_^I–VII^–C_5_^II–VII^), 70.44 (C_5_^I^), 59.16–60.42 (C_6_^II–VII^), 41.15 (C_6_^I^); HRMS–ESI (*m*/*z*): [M + Na]^+^ calcd for C_98_H_148_N_4_O_70_Na, 2523.8042; found, 2523.8125.

EDTA bis-1-adamantanylamine disodium salt, ADAdim (**4**): Adamantine (1.01 g, 6.68 mmol, 2.1 equiv) was dissolved in 30 mL of dried DMF and 10 mL of Et_3_N. The mixture was cooled to 273 K under an inert atmosphere and EDTA anhydride (0.82 g, 3.20 mmol, 1 equiv) was added portionwise. After 16 h of stirring under inert atmosphere, the solvent was removed under vacuum, and 10 mL of water was added and the solution was neutralized by HCl. The precipitate was washed with water, dried under reduced pressure then recrystallized in MeOH to obtain the diacidic compound. The diacid (814 mg, 1.46 mmol, 1 equiv) was suspended in water (10 mL) and NaOH (116 mg, 2.90 mmol, 2 equiv) was added. The mixture was sonicated for 10 min and the product precipitated by addition of 100 mL of acetone. The solid was filtered, washed with acetone and dried under reduced pressure to obtain ADAdim **4** as a white powder (*m* = 420 mg), with a yield of 52% over the two steps. The analyses are in full agreement with the literature [36]. Mp 505–506 K; ^1^H NMR (DMSO-*d*_6_, 300.13 MHz) δ 7.47 (NH, 2H), 3.37 (H_f_, 4H), 3.15 (H_d_, 4H), 2.72 (H_e_, 4H), 1.99 (H_b_, 6H), 1.91 (H_a_, 12H), 1.60 (H_c_, 12H); ^13^C NMR (DMSO-*d*_6_, 75.77 MHz) δ 172.5, 169.3, 58.8, 55.9, 52.4, 50.8, 41.1, 36.1, 29.0; HRMS–ESI (*m*/*z*): [M + Na]^+^ calcd for C_30_H_46_N_4_O_6_Na, 581.3315; found, 581.3299.
